# Medical Study

**Published:** 1837-01

**Authors:** 


Art. IV.—*Medical Study; an introductory Address delivered at the Bristol Medical School, October* 1, 1836, *at the opening of the Winter Session.* By J. A. Symonds, m.d., Physician to the Bristol General Hospital, and Lecturer on the Theory and Practice of Medicine. *Bristol,* 1836. 8vo. pp. 34.

If we were called upon to point out one thing which more than any other indicates the great increase and extension of medical knowledge among the members of our profession generally, of late years, we would mention the rise, progress, and present prosperity of the Provincial Schools, and name the many eminent men by whose talents they are supported. Among these schools that of Bristol is not the least conspicuous, and the author of the pamphlet before us is certainly one of the most eminent of the provincial teachers. It is impossible that schools established in places where the population is sufficiently large can fail to succeed, provided those appointed to teach in them are selected solely on account of their knowledge and talents; and, looking to what we know of Provincial Schools, and to the works that have of late years issued from the provincial press, we see no reason to doubt that this, the only true rule of action in such cases, has hitherto generally prevailed in them. We are sure that no one who has read the valuable contributions which the author of the present pamphlet has made to the literature of his profession, will doubt that the proper rule has been deviated from in the case of his appointment.

The introductory lecture now before us is a general exposition of the nature of the objects of medical study, of the best method of attaining these, and of the spirit that should guide the student in pursuing them. There is nothing particularly new in the details, nor indeed could much novelty be expected on such a subject; but every thing is placed in the clearest light and in the most attractive position, while the whole discourse is animated and dignified by those elevated views of the nature and duties of our profession which it is so important to impress on the mind of the student. This is the more important in the present time, when it would seem as if some men were anxious to fix the attention of practitioners on the mere financial part of our profession, to the exclusion of all considerations of higher and holier import. Not so, Dr. Symonds.

> "The instructor in medical science" (says he,) "is bound to look beyond his hearers; if his precepts are operative at all, they cannot remain with the individual; they must, by necessity, extend indefinitely; and their ministry must be productive of great happiness or of great misery." . . . "Our highest aim, however far short of it we may fall, is to fashion you into great instruments of good to mankind; and we regard a medical school, not merely as a place for learning a particular craft or calling, whereby the proficients shall gather to themselves maintenance, riches, fame, or station, according to their several kinds or degrees of ambition; but as an institution from which combatants with disease shall go forth, not as mere mercenaries, but as voluntary and ardent champions of distressed humanity." (P. 5.)

And again—

> "Our emotions, to a certain extent, are in our own power; and there is one which I would earnestly urge you to cultivate: I mean, a profound respect for your profession. Without it, you cannot long be successful. Even charlatans, who have begun with a belief in the absurdity of their pretended arts, have nourished a respect for them, from finding, that when most deceived themselves, they have been most successful in deceiving others. If this obtains where the system is itself false, how much more likely is it to hold good, when there is a real ground for conviction. Now, to acquire a feeling of reverence, if you are not already in possession of it, I think it can only be necessary to consider, what order of minds have been delightedly engaged in medical pursuits, from the earliest periods; and to observe the actual dominion which has been achieved over maladies, once the plagues of the human race, and the increased value of human life, under the improvements of our art. And I will add, that the pure and lofty aims of our profession, even if it had accomplished nothing, would alone offer sufficient demands on our veneration. Give no ear then to the idle taunts of those out of the profession: and if you find any among its ranks who dare to speak slightingly of its deeds, or doubtfully of its powers, you may safely set them down as persons who have not yet learned to avail themselves of its resources, or who have failed in their endeavours; men driven by disappointment into disaffection. Not to expect success in the treatment of disease, is, probably, the surest way of not obtaining it." (P. 28.)

We earnestly recommend this lecture to every medical student.

